# The role of body mass index, healthy eating-related apps and educational activities on eating motives and behaviours among women during the COVID-19 pandemic: A cross sectional study

**DOI:** 10.1371/journal.pone.0266016

**Published:** 2022-03-28

**Authors:** Justyna Modrzejewska, Adriana Modrzejewska, Kamila Czepczor-Bernat, Paweł Matusik

**Affiliations:** 1 Institute of Pedagogy, University of Bielsko-Biala, Bielsko-Biala, Poland; 2 Department of Psychology, School of Health Sciences in Katowice, Medical University of Silesia in Katowice, Katowice, Poland; 3 Institute of Psychology, University of Wrocław, Wrocław, Poland; 4 Department of Pediatrics, Pediatric Obesity and Metabolic Bone Diseases, Faculty of Medical Sciences in Katowice, Medical University of Silesia, Katowice, Poland; St John’s University, UNITED KINGDOM

## Abstract

The COVID-19 related lockdown made it much more difficult for people to control their eating behaviours and body weight with the methods and means they had used before. This is reflected in reports that show that eating behaviours deteriorated significantly during the COVID-19 pandemic (including in Poland). Therefore, it is important to determine what factors may be conducive to healthy eating behaviours among people with different BMI. As previous studies show, the use of healthy eating related-apps and training programs may be a protective factor against the development of unhealthy eating behaviours. Therefore, it is worth checking whether their action will be a protective factor during COVID-19. The aim of this cross sectional study was to analyse whether the current use of healthy eating-related apps and previous participation in training in this field (educational activities) as well as body mass index may play a role in eating motives and behaviours among women during COVID-19. Our final sample included 1,447 women (age: M = 31.34 ± 11.05). Participants completed: the Eating Motivation Survey, the Emotional Overeating Questionnaire, the Mindful Eating Questionnaire, socio-demographic survey and questions about healthy eating-related apps and training (educational activities). Referring to the selected significant results, our study shows that during COVID-19, the use of healthy eating-related apps alone, as well as the use of apps and prior training participation promote healthy eating motives and behaviours. It suggests that promoting the use of healthy eating applications and the acquisition of knowledge and skills in this field could be one way of shaping resources that can be effectively used to deal with crisis situations.

## Introduction

Research from most parts of the world shows that the COVID-19 pandemic has disturbed many areas of our lives and has caused numerous changes in eating behaviours [1–7]. Some authors indicate that lockdown contributed to the deterioration of eating habits (e.g. a deterioration of diet quality [4], an intensification of restrictive and binge eating behaviours [5] and more frequent snacking [7]). Among many people, COVID-19 is associated with severe psychological distress and health-related concerns [8, 9], and as the World Health Organization [10] points out, caring for mental and physical health is a very important element in building resilience during a pandemic. At the same time, it turns out that the provision of psychological and medical services due to COVID-19 has been significantly impeded [11, 12]. Therefore, in many countries it has been decided to officially implement new e-health solutions (e.g. telemedicine, teletherapy) [11]. Moreover, people have independently undertaken specific behaviours that are aimed at maintaining and/or improving health during COVID-19 and one such example is the use of fitness mobile applications [13]. Indeed, research shows that after the outbreak of the pandemic, the number of downloads of such applications increased significantly [13]. As is known, many of these applications have an educational function and significantly increase motivation and improve pro-health behaviours [14–16]. Therefore, it would be interesting to see if the current use of the healthy eating app actually may promotes healthy eating behaviours.

It is well known that how we eat can be determined by many different factors, including the state of knowledge and awareness of healthy eating [17–19]. One way to build knowledge may be to develop it through participation in healthy eating training programs. Most often, these are expected to lead to significant changes in the context of cognitive functioning (e.g. increase in knowledge and awareness), as well as to cause behavioural changes (e.g. the development of various skills related to healthy eating) [20, 21]. The effectiveness of many activities improving eating behaviours has been confirmed in studies before the outbreak of the pandemic [22–25]. Therefore, it would be interesting to know whether (next to the current use of the healthy eating app) earlier participation in training on healthy eating may be of importance in taking up eating behaviours during the COVID-19 pandemic.

The role of healthy eating-related apps (all consumer-grade apps that can support healthy eating; e.g. Fitatu, MyFitnessPal, FatSecret) and educational activities can be analysed in relation to many aspects of nutrition-related functioning because this category is very broad. This includes, among other things: (a) eating motives–refers to why people make certain food choices each day (including health- and body-related factors) [26]; (b) emotional overeating–eating excessive amounts of food under the influence of various emotions (e.g. anxiety, sadness, anger, tiredness) [27]; (c) mindful eating–is a way of eating in which people eat in response to internal cues of hunger and satiety and awareness of their physical and emotional state [28]. Moreover, at this point it should also be mentioned that numerous studies indicate that the factor that differentiates the level of eating motives and behaviours is body mass index (BMI) [26, 29–32]. These outcomes show, among other things, that in people with excess body weight, we can observe a higher level of unhealthy motives and eating behaviours (e.g. that is, more emotional and less mindful eating) compared to people with a healthy body weight [7, 26, 29–32]. Therefore, it should be taken into account in the conducted analyses.

Both healthy eating related-apps and training programs are widely available [13, 33, 34]. These can lead to improvements in many respects. Apps and training programs can result in, among others [16, 22–24, 35–40]: (a) the strengthening of body- and health-related motives of dietary intake; (b) an improvement in mindful eating; (c) a reducing in emotional overeating. Most healthy eating-related apps and training programs were prepared prior to the outbreak of the pandemic and their usefulness should now be assessed, the more so as there are indications that they may have a positive effect on diet [13, 41–43], but there has been no empirical verification of these hypotheses.

To sum up, in the current situation of significant difficulties in accessing medical services [11, 12], it seems to be particularly important to prevent obesity-related eating behaviours by having people undertake independent activities. Wang et al. [16] mentions the effectiveness of apps on independently working towards health behaviours, referring to Theory of Planned Behavior. This is because by developing the appropriate attitudes towards the behaviour, perceived behavioural control and subjective norms, we can prevent further physical and mental health complications in the long term [16, 44]. In this context, it should also be mentioned that, based on previous studies, it can be concluded that gender may be a confounding factor for the analysed aspects of functioning (e.g. significant gender differences in: eating habits [2], control or regulate eating [6], emotional eating [29]), and the group of women (due to, inter alia, a much higher risk of developing eating disorders) should be a priority group for the conducted analyses. Therefore, the aim of this study has been to analyse whether the current use of healthy eating-related apps and previous participation in training in this field (educational activities) as well as body mass index may play a role in eating motives and behaviours among women during COVID-19. We hypothesized that women with healthy weight who use healthy eating-related apps and have previous participated in training in this field will have the healthiest eating motives and behaviours.

## Materials and methods

### Participants and procedure

The Ethical Research Committee approved our cross sectional study (no. 2021/2/2E/2) and all procedures were conducted in accordance with the Declaration of Helsinki. All participants were informed about the aims of the study, and participation was anonymous and voluntary. Then they signed a consent form to participate in the study (a written consent was obtained) and completed an online survey (all data–screening and questionnaires–were collected in one measurement). Participants were not remunerated and were recruited via flyers (posted at workplace locations and universities) and an online advertisement from January to March 2021.

### Measures

#### The Eating Motivation Survey (EMS): Short version

This survey measures “why people eat what they eat” [26]. It includes 45 items and 15 subscales (eating motives): liking, habits, need & hunger, health, convenience, pleasure, traditional eating, natural concerns, sociability, price, visual appeal, weight control, affect regulation, social norms, social image. All items are rated on a 7-point scale ranging from *never* to *always*. The higher scores reflect greater eating determination by particular motives. We used only health- and body-related subscales and the Cronbach’s alpha coefficient was: α_Health_ = 0.82, α_Need & Hunger_ = 0.80, α_Weight Control_ = 0.81.

#### The Emotional Overeating Questionnaire (EOQ)

This questionnaire assesses how often (over the last 28 days) people have eaten excessive amounts of food when they feel: anxiety, sadness, loneliness, tiredness, anger, happiness, boredom, guilt and physical pain [27]. In this 9-item questionnaire, the scores range from *no days* to *everyday*. The higher the score, the higher emotional overeating. Internal-consistency coefficients were acceptable (α = 0.91).

#### An abbreviated version of the mindful eating questionnaire (MES)

This 20-item measure examines the level of two aspects of mindful eating: (a) awareness–eating while being aware of physiological and psychological experiences; (b) recognition–eating taking into account hunger and satiety [45]. Items were rated on a 4-point scale (from *never/rarely* to *usually/always*). The higher scores reflect greater mindful eating. Cronbach’s alpha coefficient was: α_awareness_ = 0.84, α_recognition_ = 0.70.

#### Socio-demographic survey and questions about apps and training

Participants completed socio-demographic variables (gender, age, weight, height, sexual orientation and ethnicity). The self-reported weight and height were used to calculated body mass index (BMI): (a) underweight: <18.5 kg/m^2^, (b) normal weight: 18.5–24.99 kg/m^2^, (c) excess weight (overweight weight and obesity): ≥ 25 kg/m^2^. Additionally, we asked: (a) Have you used a mobile application that facilitates healthy eating on a daily basis during the COVID-19 pandemic? (dummy-coded variable: *No* vs *Yes*); (b) Did you participate in any educational training about healthy eating before COVID-19 pandemic? (dummy-coded variable: *No* vs *Yes*).

### Data analysis

We used IBM SPSS Statistic version 26. To verify our hypothesis we used a two-step cluster analysis which is appropriate for sample higher than 200 and both categorical and continuous variables [46]. We conducted analysis and identified clusters based on using healthy eating-related apps, previous participation in training in this field (educational activities), and BMI. The assumptions of one-way ANOVA are not met. Therefore, a Kruskal-Wallis test was used to evaluate differences between the clusters with regard to health- and body-related eating motives (health, need & hunger, weight control), emotional overeating, and mindful eating (awareness, recognition). The Bonferroni adjusted *p*-value was applied in the post hoc multiple comparisons. The *p* value less than 0.05 was consider as significant.

## Results

### Cluster analysis of using healthy eating-related apps, previous participation in training in this field (educational activities), and body mass index

One thousand five hundred and ninety-four people volunteered to participate in the study. Our final sample included 1,447 women, because 147 participants had significant missing data gaps (screening data and/or questionnaires data were missing) and were excluded from our analysis. Women aged between 18 and 72 years and the self-reported body weight ranged from 36 to 130 kg, height from 146 to 186 cm and body mass index from 14.24 to 47.96 kg/m^2^ (Table 1).

**Table 1:** Socio-demographic characteristics of participants.

	*M* (*SD*)
**Age**	31.34 (11.05)
**Weight**	65.78 (13.48)
**Height**	166.22 (6.08)
**Body mass index (BMI)**	23.79 (4.59)
	*N* (%)
**Ethnicity** • White • Black • Asian • Mixed race • Other	• 1442 (99.65) • 0 • 0 • 3 (0.21) • 2 (0.14)
**Sexual orientation** • Heterosexual • Lesbian • Bisexual • Pansexual/Queer • Asexual • Other	• 1317 (91.02) • 28 (1.93) • 78 (5.39) • 0 • 8 (0.55) • 16 (1.11)

The five clusters were labeled, and were characterized, as follows: (a) Cluster 1 (*N* = 624): no apps, no training and normal BMI (*M* = 21.59, *SD* = 2.33); (b) Cluster 2 (*N* = 220), no apps, no training and excess BMI (*M* = 29.98, *SD* = 4.03); (c) Cluster 3 (*N* = 152): no apps, training and normal BMI (*M* = 24.84, *SD* = 5.32); (d) Cluster 4 (*N* = 82): apps, training and normal BMI (*M* = 24.02, *SD* = 5.07); (e) Cluster 5 (*N* = 369): apps, no training and normal BMI (*M* = 23.32, *SD* = 3.87). The socio-demographic clusters’ s characteristics are presented in Table 2.

**Table 2:** Socio-demographic characteristic of the five clusters.

	CLUSTER 1 (*N* = 624): apps: NO (100%) + training: NO (100%) + normal BMI	CLUSTER 2 (*N* = 220): apps: NO (100%) + training: NO (100%) + excess BMI	CLUSTER 3 (*N* = 152): apps: NO (100%) + training: YES (100%) + normal BMI	CLUSTER 4 (*N* = 82): apps: YES (100%) + training: YES (100%) + normal BMI	CLUSTER 5 (*N* = 369): apps: YES (100%) + training: NO (100%) + normal BMI	
	*M* (*SD*)					***F***
**Age**	30.22 (10.77)^a^ ^b^ ^A^	38.53 (11.83)^a^ ^¥^ ^c^ ^d^	34.14 (11.89)^b^ ^¥^ ^e^	30.17 (10.69)^c^	28.04 (8.36)^A^ ^c^ ^e^	*F*(4, 1442) = 39.60, *p* < 0.001
**BMI**	21.59 (2.33)^a^ ^b^ ^c^ ^d^	29.98 (4.03)^a^ ^e^ ^f^ ^g^	24.84 (5.32) ^b^ ^e^ ^h^	24.02 (5.07)^c^ ^f^	23.32 (3.87)^d^ ^g^ ^h^	*F*(4, 1442) = 224.33, *p <* 0.001
	*N* (%)					***χ***^***2***^ **and Cramer’s *V***
**Ethnicity** • White • Black • Asian • Mixed race • Other	• 621 (99.52) • 0 • 0 • 2 (0.32) • 1 (0.16)	• 219 (99.55) • 0 • 0 • 0 • 1 (0.45)	• 151 (99.34) • 0 • 0 • 1 (0.66) • 0	• 82 (100.00) • 0 • 0 • 0 • 0	• 369 (100.00) • 0 • 0 • 0 • 0	χ^2^_(8)_ = 5.72 Cramer’s *V* = 0.04^NS^
**Sexual orientation** • Heterosexual • Lesbian • Bisexual • Pansexual/Queer • Asexual • Other	• 562 (90.07) • 12 (1.92) • 40 (6.41) • 0 • 3 (0.48) • 7 (1.12)	• 201 (91.36) • 5 (2.27) • 9 (4.10) • 0 • 1 (0.45) • 4 (1.82)	• 137 (90.13) • 4 (2.63) • 8 (5.26) • 0 • 2 (1.32) • 1 (0.66)	• 74 (90.24) • 3 (3.66) • 4 (4.88) • 0 • 0 • 1 (1.22)	• 343 (92.96) • 4 (1.08) • 17 (4.61) • 0 • 2 (0.54) • 3 (0.81)	χ^2^_(16)_ = 9.78, Cramer’s *V* = 0.04^NS^

100% in relation to the apps and training means that 100% of participants answered "Yes" or "No" to the following questions: (a) "Have you used a mobile application that facilitates healthy eating on a daily basis during the COVID-19 pandemic?"; (b) "Did you participate in any educational training about healthy eating before COVID-19 pandemic?". Normal BMI– 18.5 ≥ BMI ≤ 24.99 kg/m^2^; excess BMI–BMI ≥ 25 kg/m^2^

^a^,^b^, ^c^,^d^,^e^,^f^,^g^,^h^–the significant (*p* < 0.001) differences between the clusters

^A^–the significant (*p* < 0.05) differences between the clusters

¥ –the significant (*p* < 0.01) differences between the clusters

NS–a non-significant.

### Comparison of the five clusters for health- and body-related eating motives, emotional overeating and mindful eating

A Kruskal-Wallis tests showed the significant effect of clusters on health- and body-related eating motives, emotional overeating, and mindful eating. Table 3 shows results of multiple comparisons.

**Table 3:** Post-hoc tests.

	CLUSTER 1 (*N* = 624): apps: NO + training: NO + normal BMI	CLUSTER 2 (*N* = 220): apps: NO + training: NO + excess BMI	CLUSTER 3 (*N* = 152): apps: NO + training: YES + normal BMI	CLUSTER 4 (*N* = 82): apps: YES + training: YES + normal BMI	CLUSTER 5 (*N* = 369): apps: YES + training: NO + normal BMI	
	*M* (*SD*)					**Post hoc**
**EMS**	*H*(4) = 159.63, *p* < 0.001					
Health	11.94 (4.30)	10.82 (4.41)	11.97 (4.68)	14.59 (4.67)	14.82 (3.88)	1 vs 2*** 1 vs 3 1 vs 4*** 1 vs 5*** 2 vs 3** 2 vs 4*** 2 vs 5*** 3 vs 4*** 3 vs 5*** 4 vs 5
**EMS**	*H*(4) = 45.28, *p* < 0.001					
Need & hunger	12.83 (3.73)	12.28 (4.25)	12.34 (4.40)	13.69 (4.28)	14.20 (3.63)	1 vs 2 1 vs 3 1 vs 4* 1 vs 5*** 2 vs 3 2 vs 4** 2 vs 5*** 3 vs 4* 3 vs 5*** 4 vs 5
**EMS**	*H*(4) = 172.63, *p* < 0.001					
Weight control	9.57 (4.16)	9.65 (4.16)	10.04 (4.50)	13.05 (3.98)	12.86 (4.07)	1 vs 2 1 vs 3 1 vs 4*** 1 vs 5*** 2 vs 3 2 vs 4*** 2 vs 5*** 3 vs 4*** 3 vs 5*** 4 vs 5
**EOQ**	*H*(4) = 10.87, *p* < 0.05					
Emotional overeating	5.56 (7.14)	7.40 (9.47)	6.04 (9.01)	6.48 (8.06)	6.31 (7.60)	1 vs 2** 1 vs 3 1 vs 4 1 vs 5 2 vs 3* 2 vs 4 2 vs 5 3 vs 4 3 vs 5^⸸^ 4 vs 5
**MEQ**	*H*(4) = 21.06, *p* < 0.001					
Awareness	30.68 (6.75)	29.25 (7.20)	31.95 (6.32)	31.78 (6.52)	31.74 (6.72)	1 vs 2* 1 vs 3^⸸⸸^ 1 vs 4 1 vs 5* 2 vs 3** 2 vs 4** 2 vs 5*** 3 vs 4 3 vs 5 4 vs 5
**MEQ**	*H*(4) = 20.78, *p* < 0.001					
Recognition	26.70 (4.53)	25.36 (4.58)	26.50 (4.64)	27.35 (4.48)	26.09 (4.57)	1 vs 2*** 1 vs 3 1 vs 4 1 vs 5* 2 vs 3* 2 vs 4** 2 vs 5^⸸⸸^ 3 vs 4 3 vs 5 4 vs 5*

EMS–the Eating Motivation Survey; EOQ–the Emotional Overeating Questionnaire; MEQ–the Mindful Eating Questionnaire; normal BMI– 18.5 ≥ BMI ≤ 24.99 kg/m^2^; excess BMI–BMI ≥ 25 kg/m^2^

* *p* < .05

** *p* < .01

*** *p* < .001

^⸸^ *p* = 0.057

^⸸⸸^ *p* = 0.073.

Overall, referring to the most significant outcomes, our results indicated that Cluster 4 and 5 only differed in terms of recognition. Moreover, both of the abovementioned clusters had significantly greater pro-healthier eating motives than Clusters 1, 2 and 3. This means that for women with a healthy body weight, using only healthy eating-related apps is as effective as using the apps and participating in training programs earlier in terms of all body- and health-related motives. The risk factor for a high level of emotional overeating and a low level of mindful eating was excess body weight, because cluster 2 was significantly different from cluster 1 and 3.

With regard to mindfulness, cluster 4 and 5 favoured a higher level in the awareness subscale compared to overweight women who did not use apps and did not develop their knowledge in training (cluster 2). Similar differences between clusters 4 and 2 were also recognized in relation to the second aspect of mindful eating–recognition. Both aspect of mindfulness is also better in Cluster 5 compared to Cluster 1.

Comparing women with excessive and normal body weight, who did not use apps and did not participate in training, we noted that in the cluster with excess body weight (next to the level of mindful eating) health subscales of EMS is significantly lower than cluster 1.

## Discussion

The COVID-19 related lockdown made it much more difficult for people to control their eating behaviours and body weight with the methods and means they had used before (e.g. exercising at the gym, visits to a health professionals). This is reflected in reports that show that lifestyle and related habits such as eating behaviours changed significantly during the COVID-19 pandemic [4, 7, 47]. Increased consumption of fast food and packaged foods, and decreased consumption of fruits and vegetables have been observed, habits that can undoubtedly result in weight gain [47]. Similar negative consequences for healthy eating have also been observed in Poland [7]. Therefore, it is important to determine what factors may be conducive to healthy eating behaviours among Polish people with different BMI, despite the existing disturbances during COVID-19. Importantly in this context, in recent times, there has been a fundamental increase in the availability of health apps [48–50], and research into their use has shown that the most popular health apps are precisely those that provide information about nutrition and weight loss [51–53]. Moreover, as previous studies show [14–16, 18, 22–25, 54], the use of healthy eating related-apps and training programs may be a protective factor against the development of unhealthy eating behaviours. Therefore, it is worth checking whether their action will be a protective factor during COVID-19.

By focusing on the most important results, our study indicates that women with normal BMI who use the healthy eating-related apps and who participated in an educational training programme (cluster 4), and those (also with a normal BMI) who only use the apps (cluster 5) have a higher level of health-promoting eating motives than those who do not use the app and did not participate in any educational training with both a healthy and an excess BMI (cluster 1 and 2), and than those (with a normal BMI) who only participated in a training programme (cluster 3). There is evidence for the effectiveness of app interventions in improving eating habits [55] and for the effectiveness of health education [56]. These interventions have resulted in intentional changes in eating behaviours (e.g., higher intake of fruits and vegetables, lower intake of calories from fat; [48]). The study of Samoggia and Riedel [15] also supports the result of our study as to the positive effect of the use of nutrition apps in changing dietary motives. Indeed, the use of apps that provide nutritional information can change eating behaviour, especially when consumers are making decisions related to their health [48].

Continuing the strand of eating motives, this outcome also shows that women who are overweight and do not use a healthy eating app and did not declare participation in any healthy eating education training (cluster 2) function worse in areas such as health- and body-related eating motives than individuals with a normal BMI who also do not use a healthy eating app and did not declare participation in any education training programme (cluster 1) or only participated in a training programme (cluster 3). Our results are related to another Polish study, which indicates that quarantine causes a change in eating habits, manifested by more frequent eating, snacking and weight change, and an important finding is that overweight and obese individuals are more susceptible to these changes [7]. The increasing prevalence of obesity has sparked interest in finding effective ways to promote healthy eating and weight management, and such functions can be fulfilled by apps [57] that provide a useful way to disseminate information, for example, about a healthy diet and nutrition [37].

Another finding from our study concerns the possible risk of emotional overeating in a group of individuals with abnormal BMIs who do not use apps and who did not participate in any educational training related to healthy eating (cluster 2). This finding is particularly concerning given that overweight individuals are prone to emotional overeating, and higher BMI is also associated with increased COVID-19-related consequences [7]. Moreover, although the comparison results for emotional overeating are not entirely clear cut, it appears that application and/or participation in training is not a sufficient protective factor for healthy eating behaviours.

In our study the people with normal BMI using the app and participating in educational training related to healthy eating (cluster 4) or only using mobile apps (cluster 5) have a higher level of mindful eating-related awareness than the group with excess BMI that do not use the apps and did not participate in any educational training (cluster 2). Similar differences were noted between clusters 2 and 4 in relation to the second assessed aspect of mindfulness—recognition. In addition, women with excessive BMI who do not use the apps and did not participate in training (cluster 2) have a lower score on both subscales of mindful eating than those who have normal BMI without the apps and training (cluster 1). Which may mean that the overweight group may be the group that needs to work harder to develop and strengthen mindful eating.

To sum up, our study shows that during COVID-19, the use of healthy eating-related apps alone, as well as the use of apps and prior training participation may promote healthy eating motives and behaviours. In the literature (on pre-pandemic studies), we read of many studies looking at interventions through apps for weight management and healthy eating [58–60], as well as for mindful eating [39]. Weight loss with the help of apps [60], self-monitoring of weight management interventions with the help of apps [61], improvement of eating behaviours through techniques of mindfulness [39], among others, have been studied, which proves that smartphone apps have many advantages. These apps, as well as various training programmes and interventions (e.g. Mindfulness-Based Eating Awareness Training [62], program based on the Health Beliefs Model [18]), promote healthy eating and weight loss, which also prevents obesity [60, 63, 64]. Moreover, validation studies to confirm the accuracy of diet and nutrition measurements that are obtained using the app have been found to be good [61]. This is also confirmed by a study conducted in Norway, which consisted of determining how diet apps and those related to physical activity affect their users [61]. The results indicated that the app was effective at promoting healthy eating and physical activity [61]. They also impacted health awareness and nutrition self-efficacy [16]. Interestingly, app use affected constructs such as attitudes toward behaviour, perceived behavioural control and subjective norms, which flow from the Theory of Planned Behavior, and enhanced behavioural intention [16]. The greater the intent, the more likely that app users will exhibit healthy behaviours [65].

Our study has limitations, the main ones being: (a) the study was a cross-sectional study; (b) all variables were measured through self-reporting; (c) recruitment of participants was based on convenience sampling; (d) too little detail about app use and/or training has been explored, (e) 147 participants were excluded from the analysis due to multiple missing data (this may distort the results obtained and the conclusions of the study may become biased). It is necessary to consider these limitations when planning further research. It will allow us, inter alia, to: (a) study the trajectory of the relationship between the use of the application and the change in eating behaviours and body weight in longitudinal measurement; (b) provide objective body weight assessment, e.g. using bioelectrical impedance analysis; (c) generate random samples; (d) incorporate data on the use of apps–better insight into how long people have been using the app, what exactly is the type of app etc.; (d) training–better insight into how long ago they underwent training, what were its components etc., (e) missing data–better insight into why some participants are not providing some data (this may help to explain, among other things, if and how participants providing the data differ from those who do not). It should also be remembered that due to the above limitations and the specificity of lockdown conditions in Poland, our results may have limited generalisability.

In addition to the implications of the limitations of our research, several further directions of research can be indicated. First, current research conducted in Belgium during the COVID-19 pandemic found that individuals who had strong social support, a favorable financial situation, and higher education made healthier dietary choices than those who did not have the above-mentioned resources [66, 67]. Therefore, in subsequent studies conducted in Poland, such factors should also be taken into account when analyzing eating motives. Second, research in France on changing food choice motives during the COVID-19 pandemic also points to the importance of mood as a factor in emotionally driven eating [1]. Regulating emotions through eating is most often associated with eating sweet foods [68], which may explain the risk of weight gain. Therefore, the next project can also be enhanced through the addition of mood measurement, estimation of changes in body weight and evaluation of dietary choices, as well as determining what features and/or components healthy eating-related apps and/or training should contain to be more effective in this regard. Moreover, in further research it may also be interesting: (a) to compare healthy eating-related apps (consumer-grade apps) with dietary assessment tools (academic apps; e.g. DietCam, and My Meal Mate) in the context of available function (e.g. feedback features, recipes, personalized nutrition and objectives) and effectiveness in maintaining healthy eating; (b) to investigate: (I) whether participants used the apps before the pandemic or deliberately started using them during the pandemic, (II) for what reasons exactly did the users use the apps (because some apps are multi-functional and can also be used for other purposes—e.g. Fitbit app can be also used for heart rate, sleep, daily steps).

## Conclusions

Our results prove that in a crisis situation such as the COVID-19 pandemic, the use of healthy eating-related apps and prior knowledge and skills gained in training may favour healthier motives and nutritional behaviour among Polish women with various BMI. It means, therefore, that promoting these activities can be one of the ways of shaping resources that can be effectively used to deal with crisis situations (when the methods used so far are not fully available, e.g. exercising at the gym, visits to a health professionals. Changes in eating attitudes and behaviours also provide great opportunities for dietitians, psychologists, educators and other health care professionals to promote and educate their patients in the context of healthy eating habits. An important message of our findings is that the use of healthy eating mobile apps and the opportunity to participate in health education programs should be an integral part of nutrition education. This is because beneficial changes in eating habits can affect life satisfaction, increase self-esteem and improve quality of life. The choice of apps should be tailored to individual needs, and future research in this area should allow their use to track actual measurement of food intake. Dissemination of the positive relationship between participation in educational programs on healthy eating and the use of related apps should improve people’s awareness and change attitudes towards proper nutrition. It is important that such activities take place as part of health promotion, health education and health prevention.
